# The Eco-epidemiology of Pacific Coast Tick Fever in California

**DOI:** 10.1371/journal.pntd.0005020

**Published:** 2016-10-05

**Authors:** Kerry A. Padgett, Denise Bonilla, Marina E. Eremeeva, Carol Glaser, Robert S. Lane, Charsey Cole Porse, Martin B. Castro, Sharon Messenger, Alex Espinosa, Jill Hacker, Anne Kjemtrup, Bonnie Ryan, Jamesina J. Scott, Renjie Hu, Melissa Hardstone Yoshimizu, Gregory A. Dasch, Vicki Kramer

**Affiliations:** 1 Division of Communicable Disease Control, California Department of Public Health, Richmond, California, United States of America; 2 Jian-Ping Hsu College of Public Health, Georgia Southern University, Statesboro, Georgia, United States of America; 3 Department of Environmental Science, Policy and Management, University of California, Berkeley, California, United States of America; 4 Lake County Vector Control District, Lakeport, California, United States of America; 5 Rickettsial Zoonoses Branch, National Center for Zoonotic, Vector-borne and Enteric Diseases, Centers for Disease Control and Prevention, Atlanta, Georgia, United States of America; Baylor College of Medicine, UNITED STATES

## Abstract

*Rickettsia philipii* (type strain “*Rickettsia* 364D”), the etiologic agent of Pacific Coast tick fever (PCTF), is transmitted to people by the Pacific Coast tick, *Dermacentor occidentalis*. Following the first confirmed human case of PCTF in 2008, 13 additional human cases have been reported in California, more than half of which were pediatric cases. The most common features of PCTF are the presence of at least one necrotic lesion known as an eschar (100%), fever (85%), and headache (79%); four case-patients required hospitalization and four had multiple eschars. Findings presented here implicate the nymphal or larval stages of *D*. *occidentalis* as the primary vectors of *R*. *philipii* to people. Peak transmission risk from ticks to people occurs in late summer. *Rickettsia philipii* DNA was detected in *D*. *occidentalis* ticks from 15 of 37 California counties. Similarly, non-pathogenic *Rickettsia rhipicephali* DNA was detected in *D*. *occidentalis* in 29 of 38 counties with an average prevalence of 12.0% in adult ticks. In total, 5,601 ticks tested from 2009 through 2015 yielded an overall *R*. *philipii* infection prevalence of 2.1% in adults, 0.9% in nymphs and a minimum infection prevalence of 0.4% in larval pools. Although most human cases of PCTF have been reported from northern California, acarological surveillance suggests that *R*. *philipii* may occur throughout the distribution range of *D*. *occidentalis*.

## Author Summary

*Rickettsia philipii*, a spotted fever group rickettsia, is the etiologic agent of Pacific Coast tick fever, an emerging tick-borne disease transmitted by the Pacific Coast tick (*Dermacentor occidentalis*). The Pacific Coast tick’s range, and thus potential for exposure to *R*. *philipii*, includes most of California, southern Oregon, and northern Baja California, Mexico. This study describes the clinical manifestations of the 14 human cases reported to date in California and the known acarological risk factors for encountering PCTF. While *Rickettsia rickettsii*, the agent of Rocky Mountain spotted fever (RMSF) is genetically similar to *R*. *philipii*, PCTF is a milder disease than RMSF; the two diseases share key clinical features such as headache and fever but differ in that PCTF presents with a localized eschar (necrotic wound) but without a petechial rash. Most case-patients have illness onset dates in the summer (peak: July and August) which is coincident with the activity period of immature *D*. *occidentalis*. Results from this study support the premise that larval and nymphal *D*. *occidentalis* are the vectors of PCTF to humans in California. While only three ticks have tested positive for *R*. *rickettsii* over decades of surveillance in California, the prevalence of *R*. *philipii* in *D*. *occidentalis* ticks averages 0.4% in larvae, 0.9% in nymphs, and 2.1% in adult ticks, indicating a higher risk of acquiring PCTF than RMSF statewide.

## Introduction

*Rickettsia philipii* (type strain “*Rickettsia* 364D”) is a spotted fever group (SFG) rickettsia closely related to *Rickettsia rickettsii*, the etiologic agent of Rocky Mountain spotted fever (RMSF) [1–3]. First isolated in 1966 from an adult Pacific Coast tick, *Dermacentor occidentalis*, collected in Monterey County, California, *R*. *philipii* has been suspected to be a human pathogen since the late 1970s [1,4]. In 2008, the first confirmed case of *R*. *philipii* infection was reported in a patient from Lake County, California [5]. Here, we propose the name, Pacific Coast tick fever (PCTF), for this rickettsiosis based on its tick vector and its geographic distribution.

Subsequent cases of PCTF in California have proven that this pathogen can cause disease in other regions of California as well [6]. To date, only five human cases of PCTF have been described in the literature, including the index case [5] and four pediatric cases [6,7]. All described case-patients presented with at least one cutaneous ulcer (eschar), and the pediatric case-patients had fever and headache. Since *R*. *philipii* is closely related to *R*. *rickettsii* and even though these agents can be distinguished by mouse typing sera [1], the strong serologic cross-reactivity of human antibodies to spotted fever group rickettsiae precludes definitive serological differentiation of PCTF and RMSF cases. Thus, while serologic testing indicated that the patients were positive for SFG rickettsiae, specific diagnosis of the etiologic agent relied upon comparative sequence analyses of rickettsial polymerase chain reaction (PCR) products from an eschar swab, a scab, or a skin biopsy [6].

*Rickettsia philipii* has been detected only in the Pacific Coast tick (*Dermacentor occidentalis*). This tick species is commonly encountered in California, southern Oregon, and northern Baja California, Mexico, and also has been associated with transmission of the agents of tularemia (*Francisella tularensis*), RMSF, and Colorado tick fever virus (a *Coltivirus*) to humans [5, 8–10]. Despite its public health importance, the phenology of *D*. *occidentalis* is incompletely known, especially the seasonality of its immature stages. The seasonality and prevalence of *R*. *philipii* infection among *D*. *occidentalis* larvae, nymphs, and adults informs the acarological risk and seasonality of disease transmission to humans.

Although RMSF is the most common SFG rickettsiosis in North America, it is rarely reported in California, with an average of only one human case per year (CDPH, Statistics and Surveillance data). Acarological data similarly indicate that *R*. *rickettsii* rarely is detected in California ticks. For example, more than 6,500 ticks of 9 species collected in California were tested for SFG *rickettsiae* to determine the risk of RMSF transmission between 1960 and 1979 [2, 9]. Not a single tick tested positive for *R*. *rickettsii*, whereas 4.2% of the adult *D*. *occidentalis* were positive for *R*. *philipii* by microimmunofluorescent typing with mouse antisera [4]. During the past decade, more than 1,000 *D*. *occidentalis*, *D*. *variabilis*, and *Rhipicephalis sanguineus* ticks from southern California were tested with molecular methods for the presence of SFG rickettsiae, but only three ticks were positive for *R*. *rickettsii*: one adult *Rh*. *sanguineus* each from Riverside [11] and from Imperial counties [12, CDPH], and one adult *D*. *occidentalis* from Orange County [10]. In contrast, in southern California, *R*. *philipii* was detected in 7.7% of adult *D*. *occidentalis* [10].

Other SFG *rickettsiae* detected in California *Dermacentor* tick species include *Rickettsia rhipicephali* [2,13] in *D*. *occidentalis* and *D*. *variabilis* [2,10] *Rickettsia bellii* [14] in *D*. *variabilis* [4] and *D*. *occidentalis* [10], and *Rickettsia peacockii* in *D*. *andersoni* [15]. These rickettsiae are not known to be human pathogens.

The overarching objective of this study was to describe the eco-epidemiology of PCTF in California. This includes characterizing the epidemiology of all confirmed cases of PCTF to date; estimating the prevalence of *R*. *philipii* infection in larval, nymphal and adult *D*. *occidentalis*; determining the seasonality and optimal habitat(s) for questing *D*. *occidentalis* nymphs in northern California; and evaluating historic tick-bite records to identify the time-of-year when humans are at greatest risk of exposure to *D*. *occidentalis* larvae, nymphs, and adults.

## Materials and Methods

### Human Diagnostic Surveillance

For suspected infection with SFG rickettsiae, physicians were encouraged to submit paired serum specimens (one taken in the first week of illness and a second 2–4 weeks later) for testing at the California Department of Public Health (CDPH) Viral and Rickettsial Disease Laboratory (VRDL). While serological tests for SFG rickettsiae are not species specific, they can still assist in the differential diagnosis of suspected cases of rickettsiosis from other febrile illnesses. For patients presenting with eschars, the eschar scab or swabs of underlying eschar tissue, were additionally requested for submission for molecular analysis [16,17,18]. Currently, genetic sequencing of swab or scab samples is required to distinguish *R*. *philipii* infections from other SFG rickettsiae such as *R*. *rickettsii*.

Rickettsial diseases are reportable in California under Title 17 California Code of Regulations as Rocky Mountain spotted fever (RMSF) or “spotted fever rickettsial disease, non-RMSF.” Suspect cases are reported to the local health jurisdiction by either physicians or laboratories, and follow-up is conducted by the local health department. Information collected in follow-up included illness onset date, signs and symptoms, laboratory test results, travel history, and factors potentially associated with exposure. Human data summarized in this study was collected as part of public health surveillance for spotted fever group rickettsiosis, reportable under California Code of Regulations, Title 17 and its use in anonymized form is exempt from internal review.

SFG rickettsiosis cases are classified as either confirmed, probable, or suspect. Following the Council of State and Territorial Epidemiologists case definition adopted by the US Centers for Disease Control and Prevention (http://wwwn.cdc.gov/nndss/conditions/spotted-fever-rickettsiosis/). Briefly, a confirmed case of SFG rickettsiosis is one with fever and any one of rash, eschar, headache, myalgia, anemia, thrombocytopenia, or any hepatic transaminase elevation and serological evidence of a fourfold change in immunoglobulin G (IgG)-specific antibody titer reactive with *R*. *rickettsii* antigen by indirect immunofluorescence assay (IFA) between paired serum specimens (one taken in the first week of illness and a second 2–4 weeks later) or detection of *R*. *rickettsii* or other spotted fever group DNA in a clinical specimen. A probable case is one that is clinically compatible and has serologic evidence of elevated IgG antibody reactive with *R*. *rickettsii* (no four-fold titer increase), and a probable case is one that has serologic evidence of exposure only.

For PCTF, a confirmed case was considered one presenting with an eschar and a positive RT-PCR test result of the eschar with confirmatory sequencing, a probable case was one with a clinically documented eschar with no testing but with a documented four-fold antibody titer increase.

### Acarological Surveillance

In addition to routine acarological surveillance in California, ticks were collected in response to suspected cases of PCTF. All ticks were identified to stage and species with standard taxonomic keys [8]. Low vegetation, leaf litter, tree trunks, rocks, and logs were flagged using 1-m^2^ white flannel flags in 37 of the 58 California counties. Based on prior knowledge, surveillance efforts focused upon ecotonal habitats, primarily the intersection of chaparral and grassland, where the adult ticks are most abundant [19]. To investigate comparative prevalence of *R*. *philipii* among life stages, all three life stages were collected in Lake County and two neighboring counties (Mendocino and Sonoma) from 2008 to 2014. All adult ticks were retained alive in snap-cap microcentrifuge tubes (Eppendorf), whereas desiccation suspectible nymphs and larvae were preserved in 70% ethanol prior to testing. Ticks were tested by CDC or at CDPH for the presence of SFG rickettsiae by PCR and comparative sequence analysis [10, 20].

To determine the optimal habitat(s) and seasonality of the nymphs, surveillance was conducted biweekly at Jack London State Park (38.3432, -122.5480) in Sonoma County from May to early September in 2009 and from July to October in 2010. Biweekly, between 10:00 and 14:00 hours (Pacific Standard Time), three biologists each spent one hour flagging two sites. Site one was a leaf-litter area in a dense oak woodland and the second site was a nearby chaparral/grassland ecotonal area (total, 6 hours flagging per day). Ticks were separated by the substrate from which they were collected (A-leaf litter; B-log; C-tree trunk; D-rock).

Focused collection of *D*. *occidentalis* nymphs was also conducted in Mendocino County the University of California, Hopland Research and Extension Center (HREC) (39.0009,-123.0829) where *R*. *philipii* was isolated from ticks in the early 1970s [2] and antibodies for SFG *rickettsiae* were detected in rodents and lagomorphs [9]. In the current study, three biologists flagged leaf litter areas in ecotonal chaparral-grassland for two consecutive days in mid-August 2012 and early September 2013. Flagging was conducted during between 08:00 and 10:00, and 18:00 and 20:00 hours, thereby avoiding the high mid-day temperatures common at this location in summer. Tick collecting was carried out along ecotones (elevation, 823 m) where black-tailed jackrabbits (*Lepus californicus*), California kangaroo rats (*Dipodomys californicus*), deer mice (*Peromyscus* spp.), and dusky-footed woodrats (*Neotoma fuscipes)* are present. Description of the vegetation and habitat sampled (Maude’s Glade) has been previously described [21].

Historic occurrence of *D*. *occidentalis* adults, nymphs, and larvae that were collected ad hoc throughout California as part of routine tick surveillance or follow-up human case surveillance were queried from a statewide tick database. Historic tick collection data were queried from a statewide collection database for the time period 1948–2014 and included 2194 separate collection efforts from 54 of 58 California counties, with collections made all years with the exception of the period 1974–1986. Ticks were collected using varied methods including removal off animals but the majority were collected using flannel 1 m^2^ tick flags. Ticks were sorted by stage and month to describe the seasonality of each life stage.

### Serologic testing and molecular detection of spotted fever group rickettsiae

#### Human testing

Human sera were tested using an indirect immufluorescent antibody (IFA) test with immunoglobulin G (IgG) reactive with *R*. *rickettsii* antigen was used as previously described [5].

Human diagnostic samples (e.g., eschars, scabs and swabs) were hydrated with 200 ul sterile PBS, and DNA was extracted using the Qiagen QIAamp DNA Blood Mini Kit (QIAGEN) [6].

#### Tick testing

Prior to extracting DNA, ticks were cleaned with a brief wash using 10% bleach followed by sterile normal phosphate-buffered saline. DNA from adult and nymphal ticks was extracted, individually, using the DNeasy Blood and Tissue kit (Qiagen). DNA from pools of fewer than 20 larvae of the same species was extracted using the same protocol.

Real-time PCR assays were used to test both human and tick specimens. From 2010 to 2014, samples were screened for a 154-bp fragment of the SFG rickettsiae outer membrane protein A (*omp*A) gene using a real-time SYBR Green PCR assay modified from Eremeeva et al. [22]. Taqman assays specific for *R*. *rickettsii* and *Rickettsia* species were added in 2013 [23]. Sequencing of a 540-bp fragment of *omp*A was performed on positive samples to distinguish *R*. *philipii* from other SFGR as previously described [10].

#### Human tick-biting records

CDPH maintains an electronic database of ticks that have been recovered from people as well as those obtained during its environmental surveillance program which catalogs tick collection records from 1900 through the present. A computerized search was conducted to determine the most common life stage of *Dermacentor* species recovered from people. The seasonality of all life stages of *D*. *occidentalis* removed from people or collected by flagging was obtained.

## Results

### Epidemiological Assessment of California PCTF Cases

**Fig 1:**
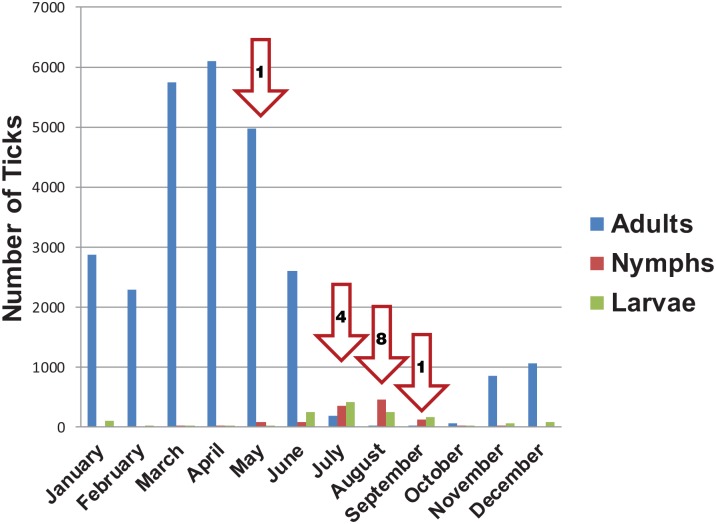
*Dermacentor occidentalis* collection records in California by month; adults were collected from 1948–2014, nymphs 1949–2014, and larvae 1969–2014. Red Arrows indicate illness onset month for human cases of Pacific Coast tick fever (numbers inside arrows represent onset of symptoms for human cases per month).

**Fig 2:**
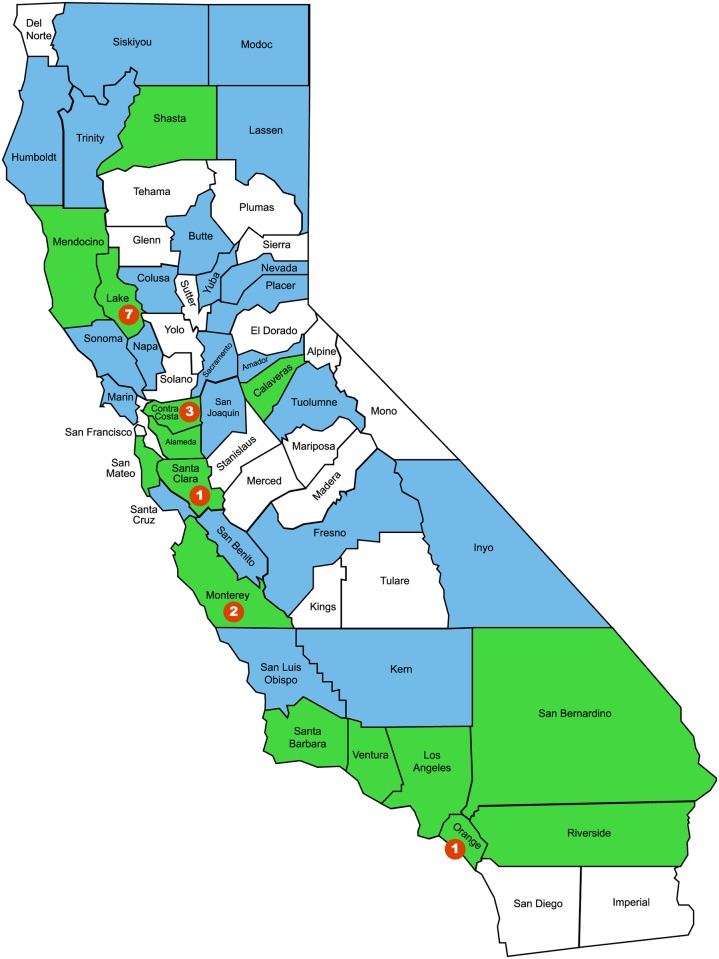
Counties where *Dermacentor occidentalis* ticks have tested positive (green) and negative (blue) for *Rickettsia philipii* (Lane et al., 1981 and Philip et al., 1981; present study). Counties with no ticks tested (white). Counties where Pacific Coast tick fever confirmed cases were acquired (red dots, number indicates number of cases), California, 2005–2014.

The 14 laboratory-confirmed human cases of PCTF were reported to CDPH by county public health departments in five counties from 2008 to 2014. Twelve (85.7%) case-patients presented in July and August, followed by one in May, and one in September (Fig 1). All case-patients were exposed to ticks in their counties of residence with the exception of two case-patients, who were likely exposed while on vacation in Lake County (Fig 2). In addition to the 14 confirmed cases of PCTF, two probable human cases were reported from Lake and Monterey counties. Although both patients presented with an eschar and were seropositive for *R*. *rickettsii*, eschar specimens were unavailable for molecular testing and thus the exact etiology could not be confirmed.

### Clinical Features of PCTF Case-Patients

**Fig 3:**
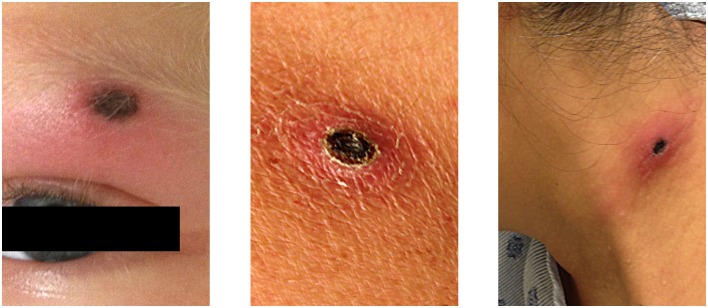
Eschars identified on the eyebrow, shoulder, and neck of three laboratory-confirmed Pacific Coast tick fever patients, California.

Signs or symptoms of the 14 confirmed PCTF case-patients included eschar (100%) (Fig 3), fever (85%), headache (79%), and lymphadenopathy (64%) (Table 1). Two (14.3%) of the 14 case-patients presented with a rash. One pediatric patient had both multiple eschars on her scalp and a petechial rash on the back of her knee, arm, dorsum of hand and ankles [7]. A male patient in his seventies presented with an eschar above his right scapula with surrounding erythema and vesicular satellite lesions. Four case-patients required hospitalization, including three with multiple eschars (2–3 eschars apiece). One pediatric patient required a two-night stay at an intensive care unit [7]. Additionally, six (43%) were male and eight (57%) were female, with a median age of 15.5 years (range 3–80). Most cases (8/14) were pediatric [6, 7]. Only three case-patients were aware of a tick bite prior to onset of illness and of these, one had onset of symptoms 6 days post tick bite; the date of tick bite for other two case patients was unknown.

**Table 1:** Comparative symptomology associated with Pacific Coast tick fever (PCTF) and Rocky Mountain spotted fever (RMSF)*.

Symptom	PCTF	RMSF***
**Eschar**	100% (14/14)	**Uncommon**
**Fever**	85% (11/13**)	100% (208)
**Headache**	79% (11/14)	72% (208)
**Lymphadenopathy**	64% (9/14)	20% (60)
**Rash**	**14% (2/14)**	92% (208)

* Includes all 14 cases reported to date in California (2008–2014)

** Temperature was not recorded for one case-patient

*** Data cited based on a previously published summary (Paddock et al., CID, 2008).

While ten case-patients presented with a single eschar, four had multiple eschars (up to three) for a total of 20 eschars described. The head and neck (35%, 7/20), the forearm (25%, 5/20), and the back and shoulder (22%, 4/18) were the most common sites for eschars. Eschars also occurred on the chest and leg. The eschars ranged in size from 1.0 × 0.5, 1.0 x 1.0, 0.6 x 0.6, to 0.2 x 1.0 cm. They generally were black in color, surrounded by erythema, and tender (Fig 3).

The clinical presentations of RMSF and PCTF share certain characteristics (Table 1). Both diseases typically present with fever, headache and lymphadenopathy. Nevertheless, the presence of an eschar versus a petechial rash can be useful in the differential diagnosis. All 14 PCTF case-patients reported in California had at least one documented eschar, a rare clinical feature in RMSF cases [24]. Similarly, the presence of a rash is typical for RMSF cases, but was only documented in 2 cases of PCTF [7].

In addition to the index case [5], serological testing with *R*. *rickettsii* antigen was performed on available sera from 13 patients (Table 2). Serological results were positive on nine (75%) of the 12 patients for whom IgM testing was performed and nine of the 13 (69%) patients for whom IgG testing was done. A four-fold or greater seroconversion in IgG titer was seen in six (75%) of the eight case-patients with paired acute (within seven days of onset) and convalescent (collected 12 or more days post onset) sera. Of the 14 case-patients, 11 were identified by scab only, two by swab of area under eschar only, and one via both scab and swab.

**Table 2:** Serological results from all human Pacific Coast tick fever case patients, California, 2008–2014.

Case*	Onset	Jurisdiction	Age (years)	SFG PCR Result (day collected post onset)	#Days serum collected post onset	Serology Results^‡^	
						R rickettsii IgG	R typhi IgG
1	July 2008	Lake Co.	80	*R*. *philipii* (13)†	6	ND	*NT*
					27	**1:2048**	*NT*
2	July 2011	Lake Co.	6	*R*. *philipii* (6)†	3	ND	ND
					7	ND	ND
3	July 2011	Lake Co.	17	*R*. *philipii* (4)†	4	ND	ND
4	Aug 2011	Contra Costa Co.	11	*R*. *philipii* (5)†	3	ND	ND
5	Aug 2011	Lake Co.	5	*R*. *philipii* (5)†	6	ND	ND
					>30	**1:128**	ND
6	Sept 2011	Santa Clara Co.	52	*R*. *philipii* (14)†	18	**>1:1024**	**1:128**
					>30	**1:256**	*NT*
7	July 2012	Orange Co.	14	*R*. *philipii* (9)†#	7	**>1:128**	ND
8	Aug 2012	Contra Costa Co.	12	*R*. *philipii* (11)†	11	**>1:256**	**1:64**
9	Aug 2012	Monterey Co.	10	*R*. *phillipii* (13)†#	13	ND	ND
					>30	**>1:1024**	*NT*
10	Aug 2012	Contra Costa Co.	3	*R*. *philipii* (9)#§	8	ND	ND
					18	**>1:256**	ND
11	Aug 2013	SF Co./Lake Co.	60	*R*. *philipii* (7)#	7	ND	ND
					19	**>1:1024**	ND
12	Aug 2013	SF Co./Lake Co.	62	*R*. *philipii* (7)#	7	ND	ND
					19	**>1:1024**	**1:128**
13	Aug 2013	Monterey Co.	57	*R*. *philipii* (7)#§	11	**1:1000**	**1:100**
					>30	**1:10000**	**1:100**
14	May 2014	Lake Co.	74	*R*. *philipii* (19)†#	19	ND	*NT*

* Cases 1–4 reported previously. Shapiro et al, 2010, Johnston et al, 2013. Cases 1–3 & 8 were confirmed at CDC.

^†^ eschar,

^#^ swab,

^§^ scab

^‡^ ND Not Detected (Titer < 1:64); *NT* Not Tested

Among the 14 case-patients, 13 were treated successfully with a 14-day course of doxycycline [5–7]. Initially, three patients also were treated unsuccessfully with ciprofloxacin (n = 1), cephalexin (n = 2), or trimethoprim/sulfamethoxazole (n = 1) prior to diagnosis. One pediatric case-patient recovered with no antibiotic treatment.

### Acarological Surveillance

During routine surveillance from 1948 to 2014, 26,735 adult *D*. *occidentalis* were collected during all months of the year, with peak abundance from March to May (Fig 1). In total, 1,115 nymphal *D*. *occidentalis* were collected from 1949 to 2015 and 952 larval *D*. *occidentalis* were collected from 1969 to 2015. In statewide collections, peak abundance was observed in July and August for nymphs and larvae (Fig 1). A total of 2194 separate tick collection events were recorded during this time period.

**Fig 4:**
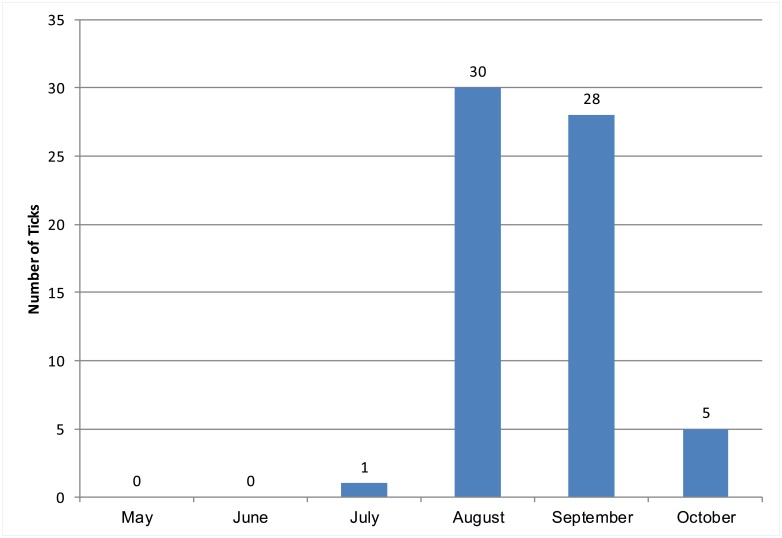
Number of nymphal *D*. *occidentalis* collected biweekly at Jack London State Park, Sonoma County, California, May-October, 2009–2010.

Focal surveillance for *D*. *occidentalis* nymphs at Jack London State Park, Sonoma County yielded 10 and 66 nymphs collected in 2009 and 2010, respectively. While nymphs were collected at this site from July through October, the largest numbers were collected in August and September (Fig 4). *Dermacentor occidentalis* nymphs were collected from leaf litter (64%), logs (21%), rocks (14%), and tree trunks (1%). While none of the *D*. *occidentalis* ticks collected at this site were positive for *R*. *philipii*, 7 were positive for *R*. *rhipicephali* (Table 3).

**Table 3:** Prevalence of *Rickettsia philipii* and *Rickettsia rhipicephali* detected in *Dermacentor occidentalis*, by county, California, 2009–2015.

County	Stage	Total tested	*R*. *philipii*	*R*. *rhipicephali*
			# positive (prevalence)	# positive (prevalence)
Alameda	Adult	190	0	12 (6.3%)
	Nymph	33	0	0
Amador	Adult	1	0	1 (100%)
Butte	Adult	16	0	1 (6.3%)
	Nymph	1	0	0
Calaveras	Adult	95	0	4 (4.2%)
Colusa	Adult	2	0	0
Contra Costa	Adult	203	0	15 (7.4%)
	Nymph	17	0	1 (5.9%)
	Larvae	7 (7 pools)	0	0
El Dorado	Nymph	31	0	6 (19.4%)
Fresno	Adult	4	0	1 (25%)
Humboldt	Adult	10	0	0
Inyo	Adult	12	0	1 (8.3%)
Kern	Adult	24	0	0
Lake	Adult	645	15 (2.3%)	59 (9.2%)
	Nymph	155	4 (2.6%)	16 (10.3%)
	Larvae	284 (47 pools)	2 (0.7%)	3 (1.1%)
Lassen	Adult	3	0	0
Los Angeles	Adult	1025	51 (5.0%)	278 (27.1%)
Marin	Adult	83	0	1 (1.2%)
	Nymph	10	0	0
Mendocino	Adult	3	0	0
	Nymph	89	1 (1.1%)	18 (2.0%)
	Larvae	3 (3 pools)	0	0
Monterey	Adult	173	0	22 (12.7%)
	Larvae	1 (1 pool)	0	0
Napa	Adult	10	0	1 (10%)
Nevada	Adult	14	0	1 (7.1%)
Orange	Adult	341	14 (4.2%)	71 (20.8%)
Placer	Adult	76	0	3 (3.9%)
Riverside	Adult	46	1 (2.2%)	2 (4.4%)
Sacramento	Adult	6	0	0
	Nymph	3	0	0
San Benito	Adult	23	0	4 (17.4%)
San Bernardino	Adult	296	1 (0.3%)	20 (6.8%)
San Joaquin	Adult	53	0	0
San Luis Obispo	Adult	31	0	3 (9.7%)
San Mateo	Adult	386	3 (0.8%)	4 (1.0%)
	Nymph	1	0	0
Santa Barbara	Adult	60	6 (10.0%)	6 (10.0%)
Santa Clara	Adult	154	5 (3.3%)	19 (12.3%)
	Larvae	1 (1 pool)	0	0
Santa Cruz	Adult	4	0	0
Shasta	Adult	83	0	2 (2.4%)
	Nymph	1	0	0
Siskiyou	Adult	48	0	1 (2.1%)
Sonoma	Adult	357	0	11 (3.1%)
	Nymph	221	0	29 (13.1%)
	Larvae	178 (12 pools)	0	4 (2.3%)
Trinity	Adult	2	0	1 (50.0%)
Tuolumne	Adult	83	0	2 (2.4%)
Ventura	Adult	2	0	0
Yuba	Adult	1	0	0
**Total**	**Adult**	**4565**	**96 (2.1%)**	**546 (12.0%)**
	**Nymph**	**562**	**5 (0.9%)**	**70 (12.5%)**
	**Larvae**	**474 (71 pools)**	**2 (0.4%)**	**7 (1.5%)**

*Minimum infection prevalence is reported in larvae.

*Rickettsia philipii* DNA was detected in *D*. *occidentalis* ticks from 15 of 37 California counties surveyed (Fig 2). In this study, 5,601 ticks were tested from 2009 to 2015 with an overall *R*. *philipii* infection prevalence of 2.1% in adults (96/4565), 0.9% in nymphs (5/562) and a 0.4% minimum infection prevalence in 474 larvae (41 larvae tested in 2 pools were positive) (Table 3). A previous study of five southern California counties (Los Angeles, Orange, Riverside, Santa Barbara, and Ventura) estimated 7.7% *R*. *philipii* prevalence in *D*. *occidentalis* [10]; in the current study, 4.9% *D*. *occidentalis* were positive from these five southern California counties.

During 2008–2014, 2,456 total ticks of all three parasitic stages (larvae, nymphs, and adults), were collected from the three counties selected for enhanced surveillance: Lake, Mendocino, and Sonoma. Both *R*. *philipii* and *R*. *rhipicephali* were detected in larvae, nymphs, and adults (Table 4). Two pools that totaled 41 larval *D*. *occidentalis* from Lake County tested positive for *R*. *philipii* (0.4% minimum infection prevalence) and 7 pools of 119 larvae from Lake and Sonoma counties tested positive for *R*. *rhipicephali* (1.5%, minimum infection prevalence). Four nymphs from Lake County and 1 from Mendocino County were positive for *R*. *philipii* (total prevalence = 1.1%) and 50 nymphs from Lake, Mendocino and Sonoma counties were positive for *R*. *rhipicephali* (prevalence = 10.7%) (Table 4).

**Table 4:** Life stages of *Dermacentor occidentalis* tested for *Rickettsia philipii and R*. *rhipicephali* by PCR, Lake, Sonoma, and Mendocino Counties, California, 2008–2014.

Stage	Number	*R*. *philipii* positive	*R*. *rhipicephali* positive
Larva	472	2 pools of 41 larvae total (0.4% MIP)	7 pools of 119 larvae total (1.5% MIP)
Nymph	468	5 (1.1%)	50 (10.7%)
Adult	1,023	16 (1.6%)	73 (7.1%)

MIP, minimum infection prevalence

The combined collections from August 2012 and September 2013 at the Hopland Research and Extension Center (HREC) in Mendocino County yielded three larvae and 88 nymphs and one adult *D*. *occidentalis* during four days of flagging. The most productive biotope for collecting *D*. *occidentalis* immature ticks was leaf litter beneath manzanita bushes (*Arctostaphylos* sp.). Whereas flagging success for immature *D*. *occidentalis* increased immediately prior to sunset (21:00 to 22:00 hr), the greatest number of ticks was collected from 07:45 to 11:00 hr (5.8 nymphs ticks per person-hour). One nymphal *D*. *occidentalis* tested positive for *R*. *philipii* (site prevalence, 1.5%). In contrast, 18 (20.5%) of 88 nymphs collected at HREC were positive for *R*. *rhipicephali*.

### Records of *Dermacentor occidentalis* Biting Humans

Most *D*. *occidentalis* ticks removed from people (n = 171) and submitted from 1971 to 2011 to CDPH for identification were nymphs (n = 117; 68.2%), followed by adults (n = 46; 26.9%; 18 males, 28 females), and larvae (n = 8; 4.7%). Nymphs bit people from June to October (peak month August), adults March to July (peak month May), and larvae May to August (peak month June).

### Rickettsia rhipicephali

*Rickettsia rhipicephali* was detected in all life stages of *D*. *occidentalis* collected from 29 counties (Table 4). The prevalence per county averaged 12.0% in adults, 12.5% in nymphs, and 1.3% minimum infection prevalence in larvae. This SFG *rickettsia* species also was detected in adult *D*. *variabilis* from Contra Costa (n = 1), Sonoma (n = 1), Fresno (n = 1) and Lake (n = 1) counties as well as in a nymph from Contra Costa county.

## Discussion

### Epidemiology

Fourteen human cases of PCTF were reported to CDPH by physicians in five California counties, from 2008 to 2014. The average number of cases (two/ year) is twice the number of RMSF cases (one/ year) reported in California during the same time period. Hence, human cases of PCTF may be more common than RMSF in California. The higher risk of PCTF is supported by the higher prevalence of *R*. *philipii* versus *R*. *rickettsii* in *Dermacentor* spp. ticks based on surveillance that dates back to at least the 1970s [2,4]. Historic records of RMSF cases reported from the 1970s include a disproportionate number of case-patients having signs or symptoms that are actually more compatible with a clinical manifestation of PCTF (i.e., 4 of 5 RMSF case reports describe an eschar and no rash; most cases were acquired in September). It has long been noted that *D*. *occidentalis* is the most abundant tick collected at those habitats where putative RMSF case-patients were exposed in California [25].

Our findings demonstrate that the months when nymphs and larvae are most active, July through September, are the same months PCTF case-patients were most likely exposed to infection. While at least 3 PCTF case-patients were aware of a tick bite, no ticks from human cases have been recovered and subsequently tested for *R*. *philipii*.

Based on the clinical presentation of the 14 PCTF human cases, the disease appears to be considerably less severe than RMSF, though some case-patients still required hospitalization. While the number of PCTF cases reported in California is low, it is interesting that the median age is 15.5 years old. It is unclear whether children are more susceptible to *R*. *philipii* or, more likely, that they are less aware of tick bites and less adept at checking themselves for ticks. Moreover, it is unclear if age or multiple eschars play a role in disease severity because our sample size is so small. Whether *R*. *rickettsii* found in California is genetically variable and can originate from different tick origins remains unknown since at least one *Rh*. *sanguineus* and one *D*. *occidentalis* tick was found to be infected with this agent and the sample genetically characterized from a RMSF case-patient was similar to types found in *D*. *andersoni* [10,11,26].

In two PCTF case-patients including the 2008 index patient, a “small black spot” was described by the patient prior at the site where the eschar later developed. Like other SFG rickettsiae, it is likely that eschars develop at the site of tick attachment [27]. In the four case-patients who presented with multiple eschars, it is unknown if each eschar was associated with a different tick bite or if these eschars were the result of a systemic infection, but the former is more probable based on what is known about other rickettsioses that present with multiple eschars [28,29]. Four PCTF cases were severe enough to require hospitalization. Alternatively, knowing that both larval and nymphal *D*. *occidentalis* bite people and can be infected with *R*. *philipii*, it is possible that a person could be exposed simultaneously to multiple tick bites without discovering the ticks, due to the small size of these ticks. Similarly, both African tick-bite fever (*R*. *africae*) and SFG rickettsiosis due to *R*. *parkeri* have been shown to result in multiple eschars in some cases [28–30]. In the case of African tick-bite fever, larval and nymphal *Amblyomma hebraeum* or *Am*. *variegatum* are the most common vector stages. The presence of multiple eschars has proved useful in differential diagnosis between sympatric SFG rickettsiosis caused by *R*. *africae* and *R*. *conorii* [29].

Serological differentiation between *R*. *rickettsii* and other SFG rickettsiae is challenging due to the high degree of cross-reactivity of human sera to related SFGR antigens. A second convalescent serum specimen is helpful in confirming the presence SFG rickettsial antibodies and guides additional testing [5]. Molecular analysis, such as PCR specifically targeting *R*. *philipii*, or nucleotide sequencing as performed here, is required to differentiate among the SFG rickettsiae.

### Acarological Findings

Information on seasonality, propensity to bite humans, and infection prevalence of *D*. *occidentalis* with *R*. *philipii* are critical to provide appropriate recommendations on how to limit exposure and avoid disease transmission. The geographic range of the Pacific Coast tick, *D*. *occidentalis*, is restricted to part of far-western North America, i.e., throughout California, southern portion of Oregon and northern Baja California, Mexico [8]. Adult *D*. *occidentalis* can and do bite people, but do so reluctantly in the experience of one of the authors (RSL). On the other hand, human-biting records from California demonstrate that the nymphs and even the larvae readily attach to people. Also, adult *D*. *occidentalis* ticks are largely inactive during late summer, the peak time of year when confirmed PCTF cases have been reported.

Previous ecological studies on *D*. *occidentalis* have focused mostly on the adult stage, which can be collected year-round with the highest abundance in March through June in chaparral-grassland ecotonal habitat in California [19, 31–33]. Nymphal *D*. *occidentalis* are rarely encountered when flagging grass but have been collected from sites with madrone or deciduous oaks [34]. Nevertheless, flagging and removing ticks from wild-caught rodents suggest that chaparral may be the preferred habitat [34–36]. Indeed, tick surveillance in northern California, such as at the University of California HREC in Mendocino County, indicated that leaf litter in chaparral, especially beneath manzanita bushes, yielded the most nymphal ticks. Collection success in these habitats was highest in the early morning and the last hour of day, suggesting that the activity period of this tick species coincides with that of its small mammalian vertebrate hosts as demonstrated experimentally [37].

At the HREC, where 1.0% of adult ticks collected in 1979 were positive for *R*. *philipii* (364D serotype) [2], an identical 1.1% (n = 89) host-seeking nymphs was PCR positive in this current study. Notably, this site is only 24 km from the exposure site of the first human case of PCTF in Lake County where one (5.2%) of 19 adult *D*. *occidentalis* was positive for *R*. *philipii* [5]. At this site, the prevalence of *R*. *rhipicephali* in both adult *D*. *occidentalis* collected in 1979 (22%) and nymphs collected in 2012–2013 (21.1%) was higher than for *R*. *philipii*.

We detected *R*. *philipii* DNA in larval, nymphal, and adult *D*. *occidentalis*. This suggests that *R*. *philipii* undergoes transovarial transmission and transstadial maintenance in ticks as occurs for many other spotted fever group rickettsiae. Transovarial maintenance is supported by our finding of 2 unfed *D*. *occidentalis* larval pools positive for *R*. *philipii*. These findings are consistent with a role for larvae as potential vectors of this rickettsia to people. Additional surveillance of this life stage is needed to determine the associated infection risk since larval *D*. *occidentalis* do occasionally bite people [8; current study]. Horizontal transmission by feeding on a *Rickettsia*-infected reservoir host or co-feeding acquisition from another infected tick are not yet demonstrated for *R*. *philipii*.

While ticks clearly serve as a reservoir for *R*. *philipii*, it is likely that mammalian reservoir hosts help to maintain infection in a region. Common host species for *D*. *occidentalis* include mammals such as jackrabbits, ground squirrels, and deer [8,19,36]. Lagomorphs such as brush rabbits (*Sylvilagus bachmani*) and black-tailed jackrabbits (*Lepus californicus*) have been shown to have a high seroprevalence to SFG rickettsia [9, 38].

*Rickettsia rhipicephali* has been detected in *Dermacentor* spp. ticks throughout California. This rickettsia was first described from the brown dog tick, *Rh*. *sanguineus* in Mississippi [13], and subsequently in *D*. *andersoni* [39], and *D*. *occidentalis* [2,4]. The prevalence of *R*. *rhipicephali* in adult *D*. *occidentalis* is estimated as high as19.4% in northern California [2,4; current study] and 27% in southern California [2,9,10; current study]. The high prevalence of *R*. *rhipicephali* in ticks throughout California and the lack of pathogenicity of this agent in guinea pigs, meadow voles, and chick embryos suggests that *R*. *rhipicephali* is an unlikely human pathogen [9]. So far, no ticks were found to be co-infected with *R*. *rhipicephali* and another SFG *Rickettsia*, such as *R*. *philipii* or *R*. *rickettsii*. It is unknown if *R*. *rhipicephali* may be similar to *R*. *peacockii* (the East Side Agent) and may serve as a protective endosymbiont to infection, possibly shielding California *D*. *occidentalis* from maintaining infections of either *R*. *rickettsii* or *R*. *philipii*. [40]

### Conclusions

Here, we propose the name Pacific Coast tick fever (PCTF) for an emerging vector-borne disease caused by *R*. *philipii* and transmitted to humans by the Pacific Coast tick, *D*. *occidentalis*. While reported cases to date have been confined to California, it is likely that people may be exposed to this disease throughout the range of this tick, which extends north into Oregon and south into Mexico. Human cases of PCTF are associated with development of an eschar, headache, and fever. Additional signs or symptoms may include lymphadenopathy, multiple eschars, and uncommonly, a rash. Over a quarter of the reported cases to date were associated with hospital visits. We posit that larval and especially nymphal *D*. *occidentalis*, not the adult stage, are the primary vector of *R*. *philipii* to humans. Unlike adult *D*. *occidentalis*, larvae and nymphs are very small and therefore less likely to be detected, thereby allowing sufficient time for transmission to occur. Furthermore, *D*. *occidentalis* larvae and nymphs are commonly collected from vegetation and off humans in summer, coincident with reported cases of PCTF. While circumstantial evidence presented here supports larvae and nymphs in the transmission of PCTF, due to transovarial and transtadial transmission, collection and testing of adult *D*. *occidentalis* ticks is a good reflection of human risk; adults are easier to collect and have a similar infection prevalence as nymphs. Based on entomological surveillance, the risk of contracting PCTF is higher than RMSF in California and exists throughout the range of the Pacific Coast tick.
